# Prenatal Diagnosis of Rare Familial Unbalanced Translocation of Chromosomes 7 and 12

**DOI:** 10.1155/2015/905946

**Published:** 2015-07-30

**Authors:** Berrin Tezcan, Foteini Emmanouella Bredaki

**Affiliations:** ^1^St George's University Hospitals NHS Foundation Trust, Blackshaw Road, Tooting, London SW17 0QT, UK; ^2^Barts Health NHS Trust, Newham University Hospital, Glen Road, Plaistow, London E13 8SL, UK

## Abstract

*Case Details*. We report rare familial unbalanced translocation of chromosomes 7 and 12, which was diagnosed prenatally at 20+3 weeks of gestation. Woman's partner had been tested in the past and was found to be a carrier of a balanced translocation; his karyotype showed a balanced reciprocal translocation of 46, XY, t(7;12)(q34;q24,32). Partner's brother had an unbalanced form of the translocation with severe learning disability. The diagnosis of the anomaly was based on two- and three-dimensional ultrasound and microarray analysis. Ultrasonography findings included fetal microcephaly and alobar holoprosencephaly, dysmorphic face (flat occiput, absent nasal bone, microphthalmia, hypotelorism, and single nostril), and hyperechogenic bowel. Genome-wide array analysis and cytogenetic results from the amniotic fluid showed unbalanced translocation in chromosomes 7 and 12 with deletion of an approximately 16.5 Mb and a duplication of 6.1 Mb, respectively, Arr 7q34q36.3(142,668,576-159,161,648)x1,12q24.32q24.33(127,708,720-133,777,560)x3, karyotype (der (7) t(7;12) (q34;q24)pat). This unbalanced translocation was due to the segregation of the father's balanced translocation. In this particular case, the recurrence of an unbalanced translocation in the subsequent pregnancies is estimated to be 20%. Understanding the individuals' phenotype in association with the gain and loss of copy number is important and can further provide us with information on that particular region of the named chromosomes.

## 1. Case Presentation

We report a case of 30-year-old Caucasian woman in her first pregnancy with a BMI of 25. The couple were nonconsanguineous. The partner had been tested in the past and was found to be a carrier of a balanced translocation that involves chromosomes 7 and 12. Partner's brother had an unbalanced form of this familial translocation between chromosomes 7 and 12 with severe learning disability. Also partner's mother was found to be a carrier of balanced translocation that involved the same chromosomes.

Woman's dating ultrasound at 11 weeks and 3 days showed an intrauterine pregnancy with a CRL (crown rump length) of 47.5 mm and a nuchal translucency of 2.0 mm. The risk of trisomy 21 was reported to be 1 : 3,300.

**Figure 1:**
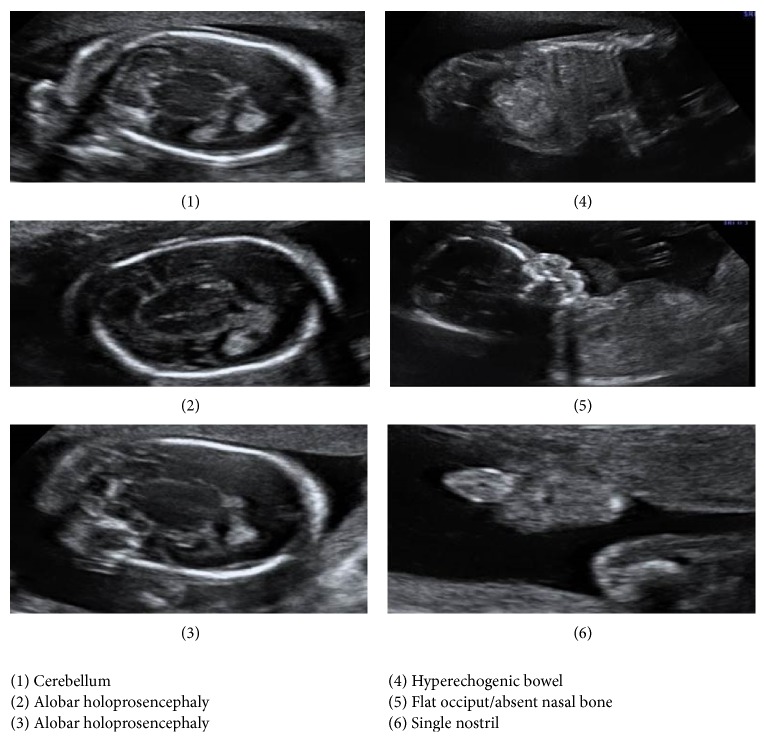
Ultrasound images of the anomaly scan performed at 20 weeks and 3 days.

A routine anomaly scan was performed by the fetal medicine specialist at 20 weeks and 3 days in view of the family history of balanced and unbalanced translocations. Anomaly scan revealed several abnormalities (Figure 1). There was marked microcephaly and alobar holoprosencephaly. Multiple facial abnormalities were detected including an absent nasal bone, microphthalmia, hypotelorism, flat profile, and single nostril. The bowel appeared hyperechogenic. All the other systems appeared normal.

The patient and her partner were counselled regarding diagnosis and poor prognosis. Due to the brain abnormalities, the prognosis was deemed to be poor with a high likelihood of miscarriage, neonatal death, handicap, and learning disability. In view of the multiple abnormalities, the possibility of an underlying chromosomal or genetic abnormality was also discussed with the couple.

The patient opted for an amniocentesis and it was performed on the same day with no complications. The patient and her partner opted for termination of pregnancy in view of associated fetal brain abnormalities. A medical termination regime was commenced and completed four days later with the delivery of a male infant weighing 320 grams. The couple opted for a full postmortem examination.

## 2. Results

### 2.1. Cytogenetic Investigations

#### 2.1.1. Parental Karyotyping

Parental karyotyping was as follows:

 Woman's blood: 46 XX.
 Female karyotype, no abnormality detected.
 Partner's blood: 46, XY,t(7;12)(q34;q24,32).
 Male karyotype with a balanced translocation between long arms of chromosomes 7 and 12 with breakpoints 7q34 and 12q24,32.

### 2.2. Fetal Amniotic Fluid

#### 2.2.1. Microarray

Fetal amniotic fluid showed Arr 7q34q36.3(142,668,576-159,161,648)x1, 12q24.32q24.33(127,708,720-133,777,560)x3.

Genome-wide array analysis indicated a copy number loss for the long arm of chromosome 7 with breakpoints at 7q34 and 7q36.3 and a copy number gain for the long arm of chromosome 12 with breakpoints at 12q24.32 and 12q24.33. This result is consistent with a deletion of approximately 16.5 Mb and a duplication of 6.1 Mb, respectively. Karyotype analysis confirmed that this result represented an unbalanced translocation product from a translocation between chromosomes 7 and 12 (der(7)t(7;12)(q34;q24)pat). This unbalanced translocation was due to the segregation of the father's balanced translocation. The array analysis result obtained was consistent with XY (male) chromosome complement.

### 2.3. Postmortem Report

**Figure 2:**
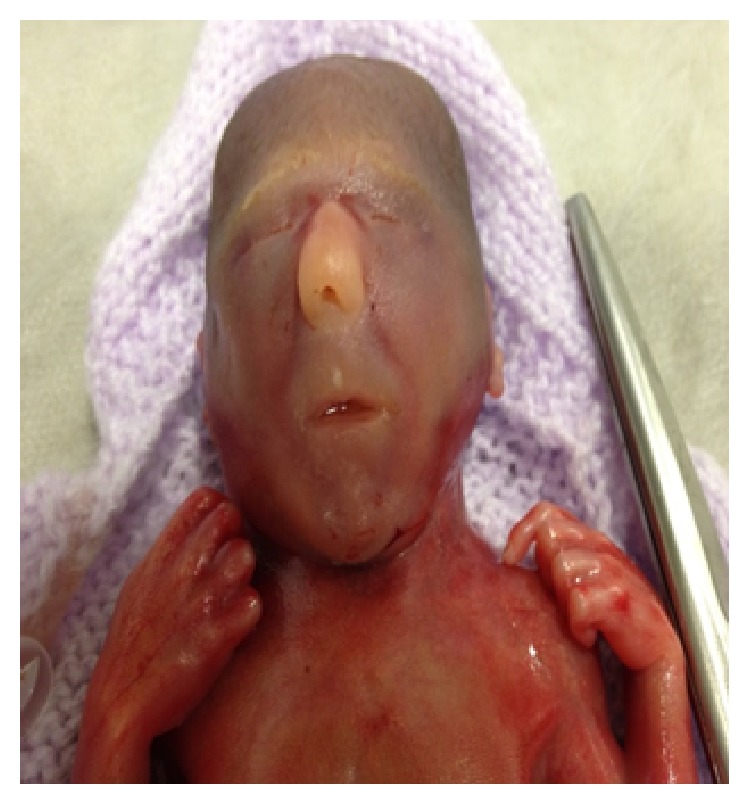
Photograph taken in the immediate postnatal period.

The body of fresh male baby was examined (Figure 2), whose measurements were consistent with 20-week gestation. The skin showed no maceration. The findings at anomaly scan were confirmed with the following additional findings: lungs were poorly lobulated; the mouth was small; the limbs and digits were normal but there was flexion of elbows and fingers; and the anus and nares were patent. External genitalia were normal male genitalia. Umbilical cord attached measured 8 × 0.7 cm and had 3 blood vessels.

## 3. Discussion

An unbalanced translocation occurs when a fetus inherits a chromosome with extra or missing genetic material from a parent with a balanced translocation. Understanding the individuals' phenotype in association with the gain and loss of copy number is important and can further provide us with information on that particular region of the named chromosomes.

It was shown that the deletion of 7q24 and 7q36 was associated with growth retardation, cleft lip and palate, and dysmorphic face [1]. This deletion also included the SHH (sonic hedgehog) gene, which was associated with holoprosencephaly. Duplication of the 12q24 was associated with multiple congenital abnormalities including holoprosencephaly, cleft palate, and dysmorphic face [2].

In one recent study, the accuracy of prenatal diagnosis for abnormal chromosome diseases by chromosome microarray technology and karyotyping was compared. In the prenatal diagnosis test, compared with karyotyping, microarray technology could identify the extra cell genetic information with clinical significance, nonparallel translocations, and aneuploidy; however, its disadvantage was that it could not identify parallel translocations and triploidy [3, 4].

In one published case of familial translocation between chromosomes 7 and 12, Ming et al. in 1980 described the familial occurrence of balanced translocation between chromosomes 7 and 12, inherited by the mother and also found in the peripheral blood of two of her five children [5], all phenotypically normal.

A literature search revealed that the t(7;12) with breakpoints at 7q31-q36 and 12p12-p13 had been reported in children with acute lymphoblastic leukemia and with myeloid disorders. Study findings suggested that ETV6 (ETS variant gene 6) rearrangements due to t(7;12) played an adverse role in myeloid disorders in children 18 months of age or younger. Therefore, children in this age group with myeloid disorders were recommended to be screened for both MLL (mixed lineage leukemia gene) and ETV6 (ETS variant gene 6) rearrangements [6].

Present case is an unbalanced translocation between chromosomes 7 and 12 that resulted in multiple fetal abnormalities mainly due to deletion of several genes. The existence of a balanced version of the translocation in the partner's genome shows a familial inheritance, with a reoccurrence risk of 20% [7]. Possible outcomes of subsequent pregnancies include (a) entirely normal karyotype, (b) balanced translocation, or (c) unbalanced translocation which may cause miscarriage or handicap [7, 8].

The couple decided to have an early prenatal testing in the form of CVS (chorionic villus sampling) in the subsequent pregnancies with a view to consider termination of pregnancy if an unbalanced type of translocation were to be diagnosed.
